# Efficacy of an extensively hydrolyzed formula with the addition of synbiotics in infants with cow's milk protein allergy: a real-world evidence study

**DOI:** 10.3389/falgy.2023.1265083

**Published:** 2023-10-09

**Authors:** Ramiro Soria, Mónica Del Compare, Marisa Sallaberry, Graciela Martín, Gustavo Aprigliano, Verónica Hermida, Mabel Carosella, Martín Gruenberg, Silvana Monsell, Paula Micone, Eugenia Maciero, Norberto Giglio

**Affiliations:** ^1^ Sanatorio Infantil San Lucas, San Miguel de Tucumán, Tucumán, Argentina; ^2^ Consultorios Externos Sanatorio Mater Dei, Ciudad Autónoma de Buenos Aires, Argentina; ^3^ Pediatras de Uriburu, Ciudad Autónoma de Buenos Aires, Argentina; ^4^ Clínica del Niño y Consultorios de Especialistas, Corrientes, Argentina; ^5^ Consultorio Pediátrico Urquiza, Ciudad Autónoma de Buenos Aires, Argentina; ^6^ Consultorios Neuropediatría Barracas, Ciudad Autónoma de Buenos Aires, Argentina; ^7^ Grupo Pediátrico Belgrano R, Ciudad Autónoma de Buenos Aires, Argentina; ^8^ Consultorio Privado, Ciudad Autónoma de Buenos Aires, Argentina; ^9^ Servicio de Tocoginecología, Hospital Durand, Ciudad Autónoma de Buenos Aires, Argentina

**Keywords:** cow's milk allergy, cow’s milk protein allergy, food allergy, extensively hydrolyzed formula, synbiotics

## Abstract

**Introduction:**

Cow's milk protein allergy (CMPA) is the most frequent food allergy in early childhood. For those infants requiring breastmilk substitutes, formulas with extensively hydrolyzed proteins (EHF), should be the treatment of choice. As there are limited data showing the progression of initial symptoms in infants newly diagnosed with CMPA who are treated with EHF with added synbiotics, the main objective of this study was to evaluate the resolution of symptoms in said infants after 4 weeks of treatment. As a secondary objective this study aimed to assess the impact of the treatment on the family's quality of life.

**Materials and Methods:**

observational, longitudinal, prospective, and multicentric real-world evidence study. The intervention phase (EHF with synbiotics) lasted 28 days and was completed by 65 patients. Treating physicians registered child´s anthropometry, Infant Gastrointestinal Symptoms Questionnaire (IGSQ-13) and CoMiSS (Cow´s Milk Allergy Symptoms Score) both at baseline and after 28 days of treatment. During treatment, caregivers reported child´s regurgitation and stools, PO-SCORAD (Patient Oriented Scoring of Atopic Dermatitis) and FAQL-PB (Family Quality of Life—Parental Burden). Data were collected using *Google Forms* and analyzed through the STATA program.

**Results:**

95.4% of the patients showed an improvement or disappearance of the overall initial symptoms after 4 weeks of treatment. Gastrointestinal symptoms improved or disappeared in 92% of patients (*p* < 0.05) while dermatological symptoms improved or disappeared in 87.5% of patients (*p* < 0.05). The median CoMiSS at baseline was 9, with 21 patients exceeding the cut-off point of 12. After 4 weeks of treatment, the median dropped to 3, and no patient exceeded the 12-cut-off point (*p* = 0.000). At baseline, patients had a PO-SCORAD of 11.5 (interquartile range 1–23) that went to 1.0 (interquartile range 1–6) at day 28 (*p* = 0.000). The treatment diminished stool frequency (*p* < 0.05), improved stool consistency (*p* = 0.004) and decreased the frequency of regurgitation in infants with CMPA (*p* = 0.01). The percentage of patients who no longer had any episode of regurgitation increased from 11% to 31% on day 28 (*p* = 0.003). At baseline, 13% of patients cried more than 3 h per day, while at day 28 that percentage dropped to 3% (*p* = 0.03). An improvement in the infants' sleep pattern was also appreciated with the treatment. At study onset, 56% of the families reported feeling very overwhelmed, a percentage that dropped to 17% after 28 days of treatment (*p* < 0.05). The small percentage of families who did not feel overwhelmed at study onset (17%), grew to 43% on day 28 (*p* < 0.05).

**Conclusions:**

The use of an EHF with synbiotics for the management of infants diagnosed with or suspected to have CMPA suggested a good safety profile, an adequate infant growth, and improvement of overall, gastrointestinal, and dermatological symptoms. It also suggested a lower daily frequency of regurgitations and stools, and an improvement in stool consistency, sleeping pattern, and quality of life of the infant and his family.

## 1. Introduction

Food allergies are a frequent consultation in pediatrics and one of the most frequent in early childhood is cow's milk protein allergy (CMPA) (1, 2). The diagnosis of CMPA tripled in the last decade, with a prevalence reported between 1.8 and 7.5% (1, 3, 4).

Breastfeeding is the most complete alternative for infants with CMPA to adequately grow and develop, with a milk protein elimination diet being the treatment of choice for the control of clinical manifestations (4). If the infant requires breastmilk substitutes, a hypoallergenic formula should be given. These can be formulas with extensively hydrolyzed proteins (EHF), or elemental formulas based on free amino acids (AAF). EHF are the first-line treatment for mild and moderate forms of CMPA (4).

As it has been shown that an alteration of the intestinal microbiota is involved in the development of CMPA (5–7), in recent years, this dietary management has gone from being passive (elimination diet to alleviate symptoms) to being proactive, using prebiotics, probiotics and synbiotics that can actively modulate the immunological system through the microbiota (8–10). It has recently been reported that the use of hypoallergenic formulas with specific synbiotics resulted in a sustained improvement in gut microbiota composition (11).

Finally, it is important to consider that, beyond its clinical symptoms, CMPA implies a much broader burden, ranging from a decrease in the quality of life of families and infants with this condition (12) to a significant economic burden on health systems (13).

For all the above reasons, the main objective of this study is to evaluate the progression of symptoms in infants diagnosed with or suspected to have CMPA after 4 weeks of treatment with an EHF with synbiotics. As secondary objectives, the study intends to evaluate the impact of treatment on the family's quality of life after 4 weeks of treatment and to describe the use of health resources up to one year of life.

## 2. Materials and methods

### 2.1. Study design

Observational, longitudinal, prospective, and multicenter real-world evidence (RWE) study. The intervention phase (single arm) lasted 28 days. To obtain data on the use of healthcare resources up to one year of life, an analysis of medical records (hospital visits, infections, medications used) was performed when the individuals reached one year of age. The study was conducted between June 2021 and December 2022 in 10 sites of 3 regions in Argentina. The approval of the Independent Ethics Committee for Trials in Clinical Pharmacology of the Foundation for Pharmacological and Drug Studies (FEFYM) “Luis María Zieher” was obtained. The study was conducted in accordance with Good Clinical Practices and the Declaration of Helsinki. The parents of all recruited patients signed a written informed consent.

### 2.2. Population

Infants with presumptive or confirmed diagnosis of CMPA, who had, as their first indication, an EHF for a minimum period of 28 days. The EHF used in this study was Nutrilon Pepti Syneo® (Nutricia, the Netherlands), a nutritionally complete food for specific medical purposes with hydrolyzed whey protein, long-chain fatty acids and synbiotics (GOSsc/FOSlc and *Bifidobacterim breve M-16V*), intended for the dietary management of CMPA in infants from birth, either as a sole source of nutrition or as a supplement to breastfeeding and/or complementary foods. The initial evaluation and subsequent follow-up of each participant was performed according to the usual practices for patients with suspected CMPA.

### 2.3. Inclusion and exclusion criteria

Patients with presumptive or confirmed diagnosis of CMPA who were less than or equal to 8 months of age, with parents already feeding their child with formula, were included. Infants who had already been previously fed hypoallergenic formulas, who suffered from severe CMPA thus requiring an AAF, or who had contraindications for consuming synbiotics (short bowel, parenteral nutrition, post pyloric feeding, central venous catheter and/or immunocompromised infants) were excluded. For follow-up at one year of life, all infants who had completed the 28 days of initial treatment were included. Children who subsequently required an AAF and those for whom data could not be extracted from medical records were excluded from this analysis.

### 2.4. Data collection and statistical analysis

For data collection, Infant Gastrointestinal Symptoms Questionnaire (IGSQ-13) (14) and Cow's Milk Allergy Symptoms Score (CoMiSS) (3) had to be completed by the treating physicians, while Patient Oriented Scoring Atopic Dermatitis (PO-SCORAD) (15) and Family Quality of Life—Parental Burden questionnaire (FAQL-PB) (16) had to be completed by the patient's families.

#### 2.4.1. CoMiSS

The CoMiSS assesses 5 clinical domains (crying, regurgitation, stools, respiratory symptoms, and skin signs), with total scores ranging from 0 to 33 (3). A score of 5 is considered normal in healthy infants under 6 months of age (17).

#### 2.4.2. IGSQ-13

The 13-item IGSQ index score assesses infants' GI-related signs and symptoms observed by parents over the previous week in 5 domains: stooling, spitting up/vomiting, flatulence, crying, and fussiness. Items were scored on a scale of 1–5, with higher values indicating greater GI distress. The total IGSQ score was calculated by summing item responses. Thus, the possible range in scores was 13–65, where a score of 13 indicated no GI distress at all and a score of 65 represented extreme GI distress (14). In addition, daily stool frequency and stool consistency (Brussels Infant and Toddler Stool Scale) were also recorded at the same time points (18).

#### 2.4.3. PO-SCORAD

This tool rates severity (from none to extreme) of dryness, erythema, edema, oozing, scratches, skin thickening, as well as indicating sleep quality and crying frequency on visual analog scales (ranging from 0 [none] to 10 [unbearable]). The numeric value from the tool indicates a score ranging from 0 (no skin affected and no symptoms) to 103 (entire body affected and extreme symptoms) (15).

#### 2.4.4. FAQL-PB

The FAQL-PB Scale is a 17-item instrument. It utilizes a 7-point Likert scale ranging from 1 (not troubled) to 7 (extremely troubled). Questions include issues concerning going on vacation, social activities and worries and anxieties over the previous week. The number circled for each question is summed to provide a total continuous score with a higher score indicating greater burden on the family. Scores can range from 17 to 119 (16).

#### 2.4.5. Use of health resources

To obtain data on the use of health care resources up to one year of life, an analysis of medical records (hospital visits, infections, medication use) was performed when the individuals reached one year of age.

#### 2.4.6. Statistical análisis

**Figure 1:**
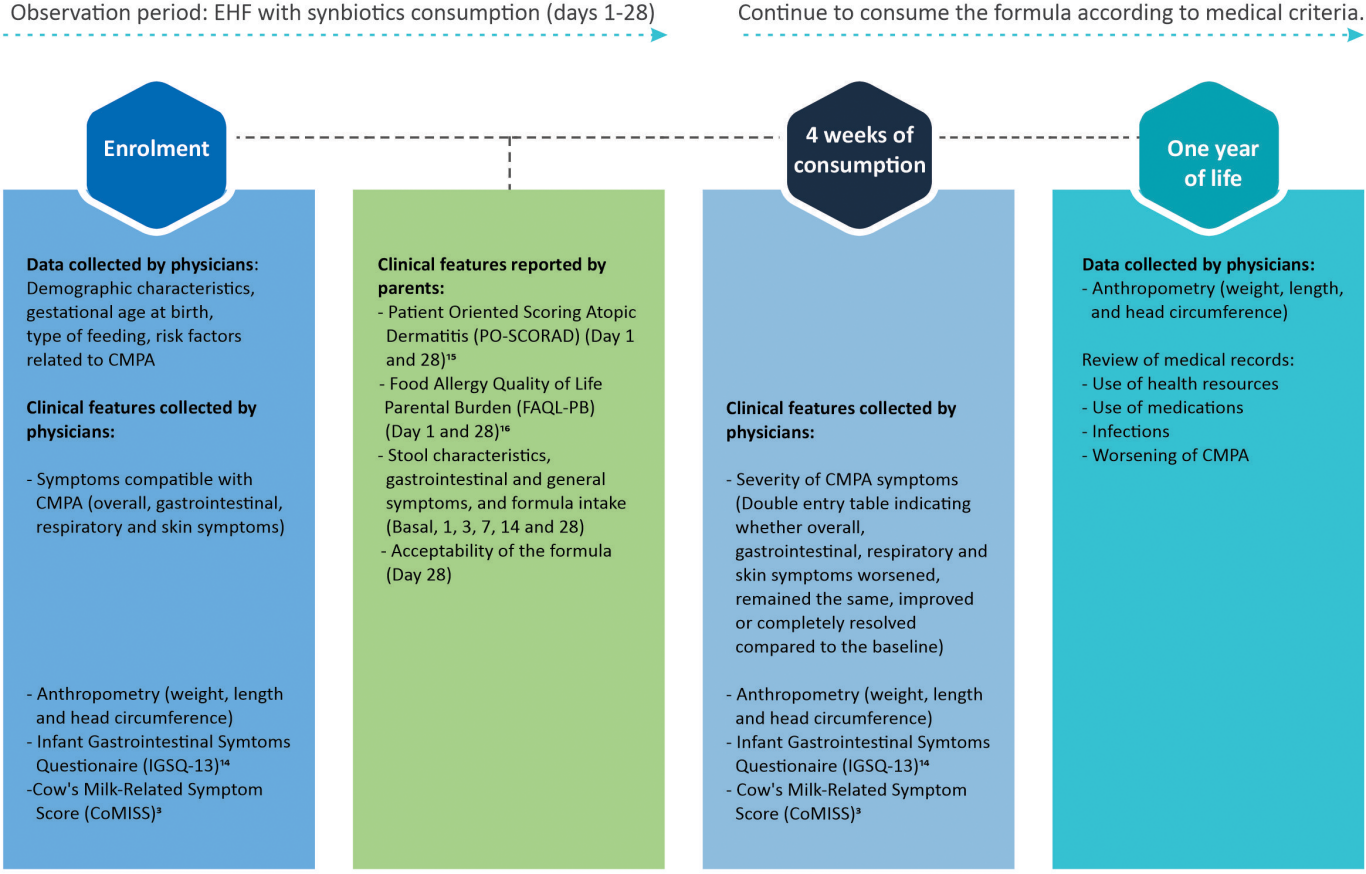
Study design.

Data were collected using Google Forms and analyzed through the STATA program. The detail of the study design can be seen in Figure 1. Data were described as frequency and percentage, for categorical variables. For numerical variables, mean and standard deviations were used for normal distributions while median and interquartile interval were used for non-normal distributions. To assess the differences from baseline, comparisons were made using Wilcoxon signed rank test for continuous variables with nonparametric distribution and χ^2^ test for categorical variables. Statistically significant differences were considered to exist when the *p*-value was less than 0.05 (*p* < 0.05).

## 3. Results

**Figure 2:**
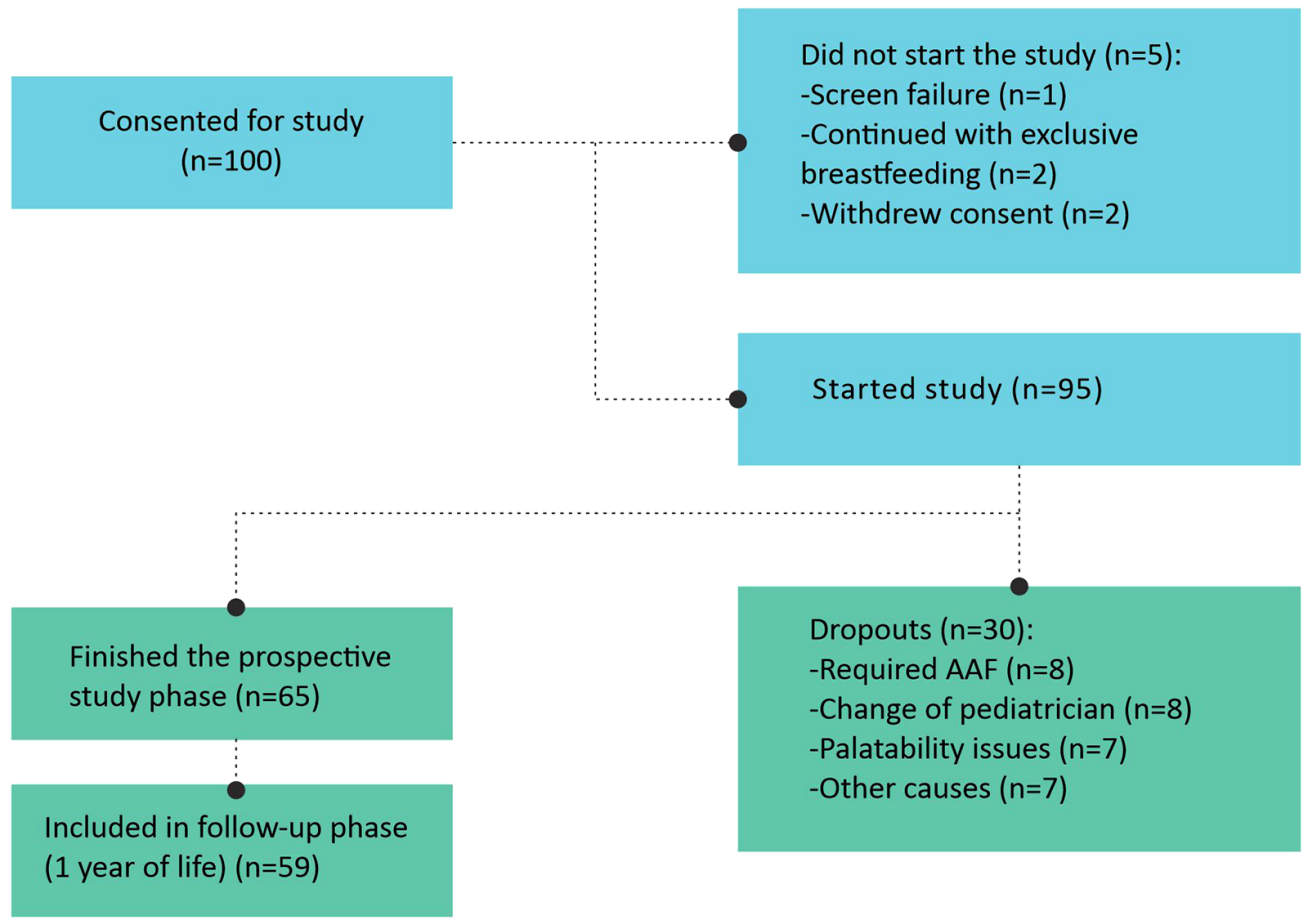
Recruitment flow.

The 28-day treatment with an EHF with synbiotics was completed by 65 patients. In 59 of them, data regarding the use of healthcare resources could be collected at one year of life. The recruitment flow can be seen in Figure 2.

Demographic characteristics of the population, route of delivery, type of feeding at study onset and family history of atopy are shown in Table 1.

**Table 1:** On the left: demographic characteristics of the population, route of delivery and type of feeding at study onset. On the right: family history of atopy (*n* = 65).

Population characteristics		Family history of atopy	*n* (%)
Sex^a^			
Female	32 (49%)	None	39 (60.00)
Male	33 (51%)		
Age at admission (in months)^b^	3.2 ± 1.98 (0.5–7)	Mother	11 (16.92)
Gestational age at birth (in weeks)^b^	38 ± 2 (31–41)	Father	7 (10.77)
Route of delivery^a^			
Vaginal	16 (25%)	Siblings	1 (1.54)
C-section	49 (75%)		
Type of feeding at study onset^a^			
Mixed feeding	51 (78%)	2 or more relatives	7 (10.77)
Exclusive formula	14 (22%)		

^a^ *n* (%).

^b^ Average ± SD (range).

### 3.1. Main outcome: clinical efficacy and symptoms resolution after 28 days of treatment with an EHF with synbiotics

At baseline, 96.8% of the infants presented gastrointestinal symptoms (60% along with dermatological symptoms, 16.9% with respiratory symptoms, 9.2% with both dermatological and respiratory symptoms, and 10.7% presenting exclusively gastrointestinal symptoms). The remaining 3.2% presented dermatological and respiratory symptoms.

The progression of these symptoms after 4 weeks of treatment with an EHF with synbiotics is shown in Table 2.

**Table 2:** Symptoms progression after 28 days of treatment with an EHF with synbiotics (*n* = 65).

	Disappeared or improved *n* (%)	Stayed the same or got worse *n* (%)	*P*
Gastrointestinal symptoms	58 (92%)	5 (8%)	*p* < 0.05
	CI 95% 85–98	CI 95% 1.4–14.5	
Dermatological symptoms	41 (87.5%)	6 (12.5%)	*p* < 0.05
	CI 95% 77–96	CI 95% 2.8–21.1	
Respiratory symptoms	12 (63%)	7 (37%)	*p* = 0.1
	CI 95% 41–85	CI 95% 15–59	
Overall symptoms	62 (95.4%)	3 (4.6%)	*p* < 0.05
	CI 95% 89–100	CI 95% 0.2–10.2	

**Figure 3:**
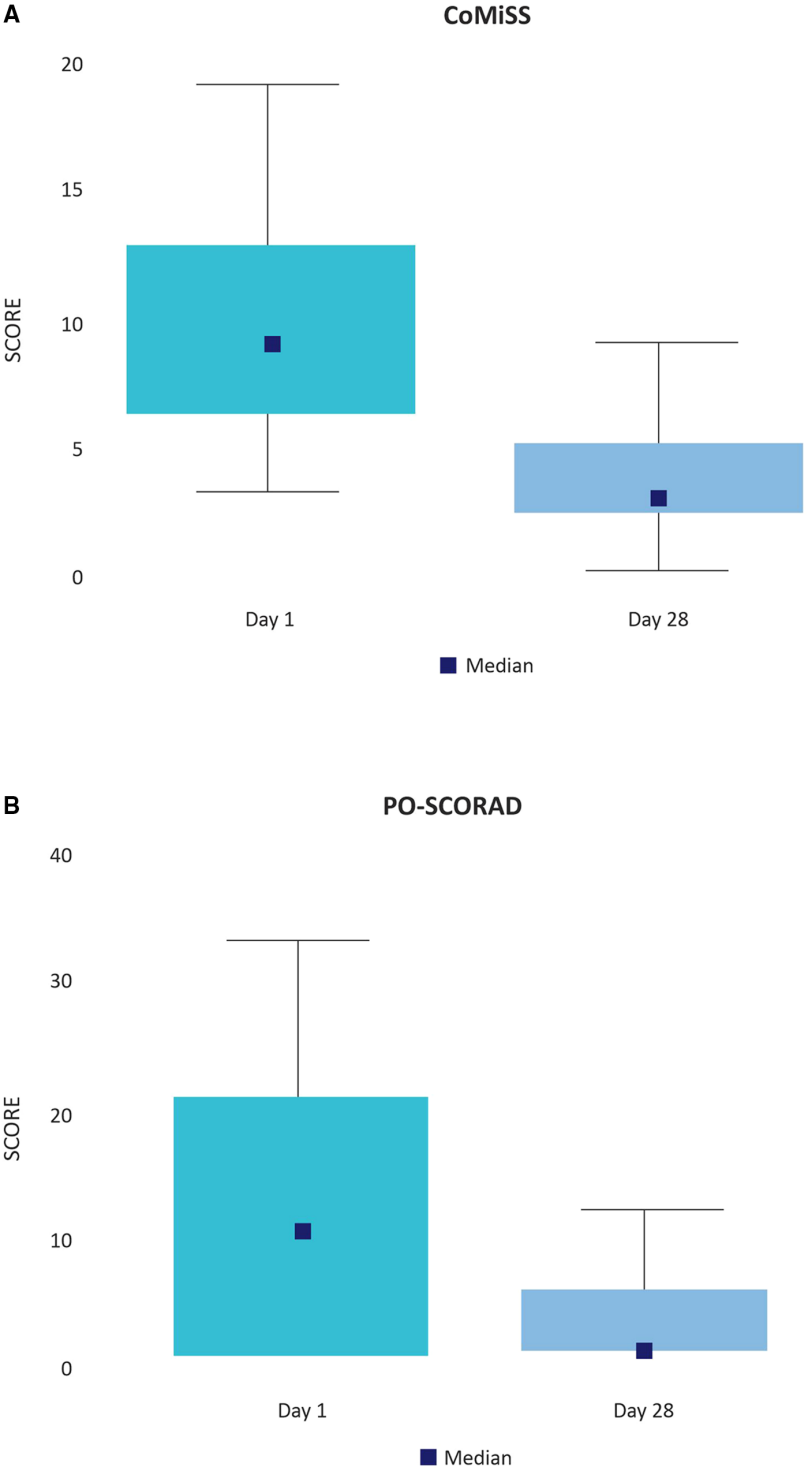
(**A**) CoMiSS (days 1 and 28). Data collected by physicians. (**B**) PO-SCORAD (days 1 and 28). Data collected by caregivers.

At baseline, 21 patients showed a CoMiSS greater than or equal to 12. At the end of the treatment, no patient exceeded it. Figure 3A shows that the median CoMiSS at study onset was 9, decreasing significantly to 3 with treatment (*p* = 0.000).

Patients included in the study had mild eczema, judging by a median PO-SCORAD at baseline of 11.5 (Interquartile range 1–23). With the treatment the median PO-SCORAD dropped to 1.0 (Interquartile range 1–6) on day 28 (*p* = 0.000) (Figure 3B).

### 3.2. Secondary outcome: the infant's and the family's life quality

**Figure 4:**
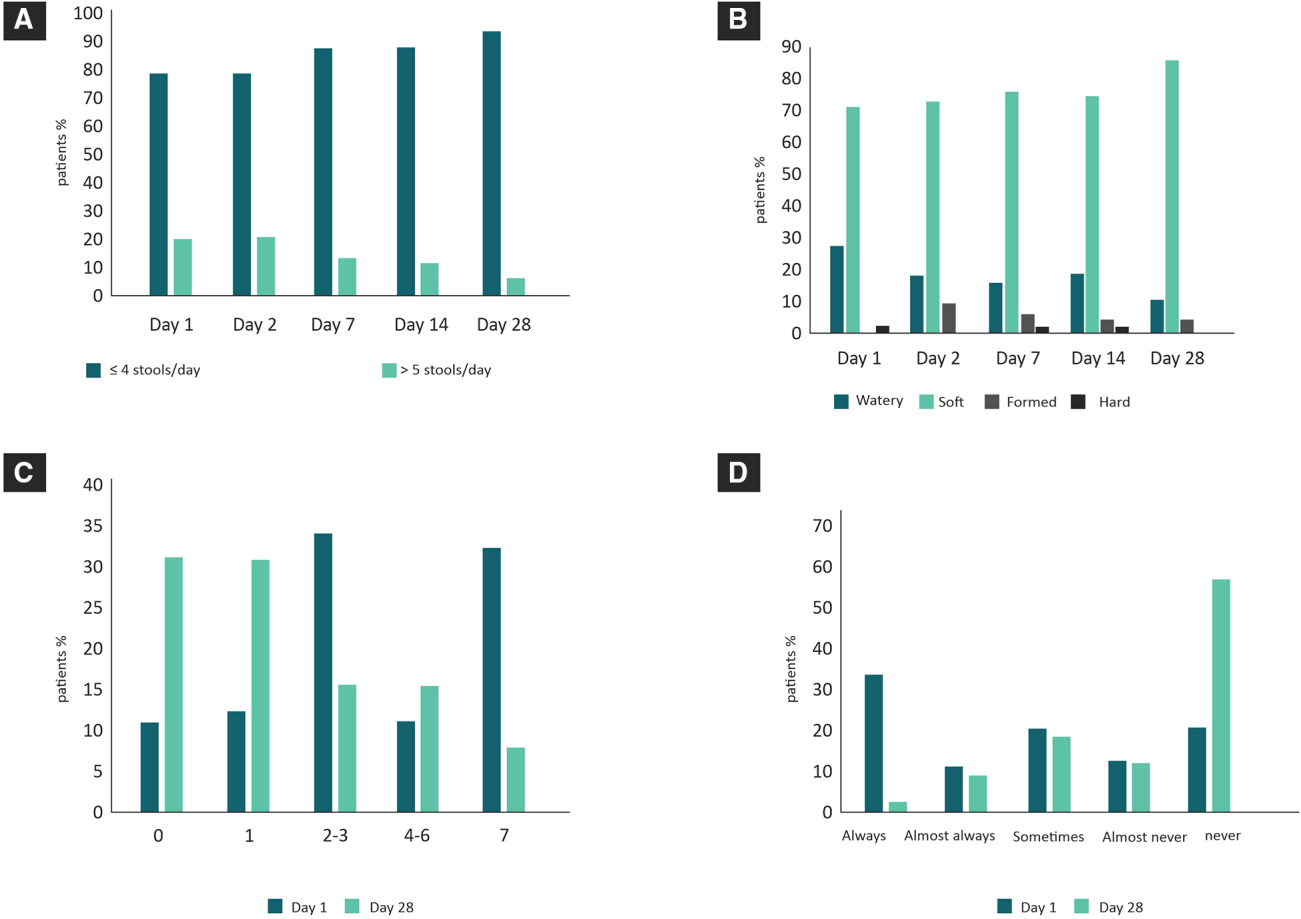
Changes in infants' stools and regurgitation with treatment (data collected by caregivers). (**A**) Stool frequency during treatment. (**B**) Stool consistency during treatment. (**C**) Number of daily regurgitations (days 1 and 28). (**D**) Frequency of infants' discomfort that was attributed to regurgitation by their caregivers (days 1 and 28).

Figure 4 shows the changes in infants' stools and regurgitation with treatment, as well as the impact of these modifications on the quality of life of their caregivers. The percentage of infants with more than 5 daily stools decreased throughout treatment between day 1 and day 28 (*p* < 0.05) (Figure 4A). Regarding consistency, 28% of parents reported watery stools on day 1, decreasing to 10% on day 28 (*p* = 0.004) (Figure 4B).

Figure 4C shows that on day 1, 43% of patients had 4 or more daily regurgitations. After 28 days of treatment, this percentage dropped to 22% (*p* = 0.01). Within this group, the percentage of patients who had more than 7 regurgitations per day changed from 32% at baseline to only 7% on day 28 (*p* = 0.0003). The percentage of patients who no longer had any episodes increased from 11% (day 1) to 31% on day 28 (*p* = 0.003).

Consistent with the above data, 34% of parents reported that their children “were always uncomfortable due to regurgitation” at the beginning of treatment, a percentage that dropped to 2% on day 28 (*p* = 0.000). On the other hand, only 20% reported not being uncomfortable on day 1, while 58% reported that they never felt uncomfortable on day 28 (*p* = 0.000) (Figure 4D).

Other relevant aspects for the quality of life of infants and their caregivers are those related to sleeping and crying. At baseline, 13% of patients cried more than 3 h per day, while on day 28 that percentage dropped to 3% (*p* = 0.03). Children who, according to their parents, did not cry due to irritability changed from 31% at baseline to 49% on day 28 (*p* = 0.03). Likewise, an improvement in the infants' sleeping pattern was appreciated with the treatment (not shown).

Table 3 shows the impact on the family's quality of life that represents an infant with CMPA and how it improves significantly with treatment. At study onset, 56% of the families reported feeling very overwhelmed, a percentage that dropped to 17% after 28 days of treatment (*P* < 0.05). The small percentage of families that did not feel overwhelmed at study onset (17%) grew to 43% on day 28 (*p* < 0.05).

**Table 3:** Impact of CMPA and its treatment with an EHF with synbiotics in the patient's family quality of life (*n* = 59).

In the last week, how overwhelmed did you feel about your child's CMPA?	Day 1 *n* (%)	Day 28 *n* (%)	*P*
Not overwhelmed/ almost not overwhelmed	10 (17%)	25 (43%)	0.02
Somewhat overwhelmed/moderately overwhelmed	16 (27%)	24 (40%)	0.13
Very overwhelmed/quite overwhelmed	33 (56%)	10 (17%)	<0.00

Data extracted from the FAQL-PB (Family Quality of Life—Parental Burden questionnaire) (16).

The median FAQL-PB score at baseline was 49 (Interquartile range 27–64), dropping to 30 (Interquartile range 18–54) with the treatment on day 28 (*p* = 0.02).

### 3.3. Secondary outcome: use of healthcare resources up to one year of life

More than half of the infants (52%) did not require any consultation to the emergency service until one year of life, while only 6 infants (10.17%) required more than 5 consultations. Of these, 2 patients had to be hospitalized for reasons unrelated to their CMPA.

42.3% of the infants did not require pharmacological medication during follow-up. Only 12% (7 patients) required additional drug treatment for their allergic symptoms, 34% used analgesics and/or antipyretics, and 24% required antibiotics.

Less than half of the infants (*n* = 29; 49%) presented at least one infectious condition: 2 presented gastrointestinal infections, 18 presented respiratory infections, 3 a combination of both, and 6 patients presented other infections.

### 3.4. Other relevant aspects

When establishing a nutritional treatment for CMPA it is also important to demonstrate an appropriate infant growth and to ensure a proper compliance with the treatment.

All the subjects included in this study presented anthropometric measures (height, weight, and head circumference) within the WHO growth chart percentiles for their sex and age. The average anthropometric measures can be seen in Supplementary Table S1.

Considering some issues of daily family management that contribute to treatment compliance, 98.5% of the caregivers found it easy to prepare the formula, and the acceptance by the infants was 69%. According to the appreciation of the caregivers, 66% of the infants seemed to enjoy it (not shown).

## 4. Discussion

### 4.1. Demographic characteristics, family history of atopy, type of delivery and feeding

The mean age of patients who entered the study with a diagnosis or suspicion of CMPA agrees with international (10) and local (19) reports. It is known that food allergy is a complex immune disorder caused by specific genetic variants in combination with environmental and nutritional exposures (20). In this context, both the high rate of C-section and the low rate of exclusive breastfeeding appreciated in our sample have been shown to interfere with the homeostasis of the intestinal microbiota and to be determinants of dysbiosis (21, 22), which is considered one of the main responsible for the increasing incidence of allergic disorders (23–25). The family history of atopy present in the recruited infants is also a risk factor associated with CMPA. It is known that the existence of a history of atopy in a sibling increases the risk of allergic disease by 25%–30%, in one parent increases it by 20%–40% and in both parents by 40%–60% (26, 27).

### 4.2. Main outcome: clinical efficacy and symptoms resolution after 28 days of treatment with an EHF with synbiotics

More than 95% of the infants showed complete improvement or resolution of CMPA symptoms with treatment, confirming the previously reported efficacy of the formula (10). Although 92% of the gastrointestinal symptoms and 87.5% of the dermatological symptoms evolved favorably, only 63% of the respiratory symptoms showed this positive outcome. From an epidemiological point of view, as the study was conducted during autumn and winter, an increase in respiratory symptoms not associated with CMPA but with seasonal viruses may have been appreciated. This is partially confirmed by the results of nasopharyngeal swabs that showed the presence of respiratory syncytial virus in some infants.

The treatment efficacy is also evidenced by the improvement of both the CoMiSS (captured by treating physicians) and the PO-SCORAD (captured by caregivers). Previous reports have shown similar results in infants with CMPA receiving an EHF with GOS, FOS and *Bifidobacterium breve M-16V* (10, 28, 29).

A multicenter, double-blind, placebo-controlled study, in which 90 exclusively formula-fed infants with atopic dermatitis were randomly assigned to receive an EHF with or without synbiotics for 12 weeks, showed that both treatments improved atopic dermatitis, as assessed by the SCORAD index. Although no significant differences were observed between the consumption of an EHF with or without synbiotics in the subgroup of patients with non-IgE-mediated atopic dermatitis, the differences were significant in favor of the consumption of EHF with synbiotics in the subgroup of patients with IgE-mediated atopic dermatitis (28).

On the other hand, it has also been reported that the treatment with EHF with synbiotics enhanced the management of infants with non-IgE-mediated CMPA who were already established on EHF without synbiotics, showing a significant improvement in atopic dermatitis in those patients with severe baseline symptoms (PO-SCORAD reduction from 34.7 to 18.2) (10).

### 4.3. Secondary outcome: the infant's and the family's quality of life

Parents or caregivers of infants with food allergies reported a lower quality of life, due to the social and emotional impact they suffer, and referred high levels of anxiety and stress (30). In our study, parents reported an improvement in their quality of life after 28 days of receiving an EHF with synbiotics. This is in line with was previously reported by Hubbard et al., who also showed a significant reduction in the FAQL-PB score between baseline (mean 30.9) and after 4 weeks of treatment with an EHF with synbiotics (mean 22.6) (10).

Non-IgE-mediated CMPA can present a wide range of gastrointestinal manifestations, including vomiting, regurgitation, or diarrhea (31). Although a randomized clinical trial comparing the consumption of an EHF with and without synbiotics showed that stool consistency was significantly softer in the group that consumed the formula with synbiotics without finding significant differences in relation to stool frequency (28), our results showed that a 28-day treatment with an EHF with synbiotics significantly improved stool consistency but also decreased the number of daily regurgitations and stools. These results were in accordance with the perception of caregivers, who reported that the discomfort that these symptoms generated before treatment decreased substantially after treatment, indicating a substantial improvement in both infants and caregivers' quality of life. For the correct interpretation of these results, it is important to consider two aspects: time and feeding. On the one hand, at least partially, the improvement of these symptoms could have occurred just over time and could not be completely attributed to the treatment. On the other hand, both the frequency and consistency of stools also depended on whether the patient is being breastfed or not. In the present study, we were unable to carry out a subgroup analysis to specify whether the decrease in the frequency and consistency of stools is seen differentially in those infants of mothers who feed their children with breast milk and formula or exclusively with formula.

Other aspects that substantially modified the infant's and the family's quality of life were the sleeping pattern and the crying attributed to irritability. There is a close relationship between food allergy and sleep disorders; an association that has increased in recent times (32). Our data indicated an improvement in the sleeping pattern of infants with CMPA after treatment, reflected both in the increase of those patients who reached what caregivers consider a normal pattern and in the lower percentage of patients with nocturnal awakenings.

Regarding inconsolable paroxysmal crying, it can often be a sign of an underlying medical condition and it may be the first manifestation of a food allergy (33). In our study, the positive evolution of CMPA symptoms led to a significant improvement in the irritability of infants, which was reflected in the decrease in the number of hours of crying after treatment and in the decrease in the crying perceived by parents as related to irritability.

### 4.4. Secondary outcome: use of healthcare resources up to one year of life

Given that the EHF evaluated in the present study has as a distinctive characteristic the addition of synbiotics with potential modulating effect on the immune system (7), and since the management of an infant with CMPA represents a significant burden for the health system in terms of the use of health resources and medical consultations (10), our study evaluated the patient's medical records at one year of life to describe the number of hospital visits, infections and medication used.

In a recent study, hospital visits and prescriptions of medications were collected from hospital medical records and caregivers, during the 6-months before, and 6-months after EHF with synbiotics treatment initiation. In said study, significant reductions in the mean number of overall hospital visits required by infants were observed in the 6 months after the treatment with EHF with synbiotics. The authors also showed a significant decrease in prescriptions for gastrointestinal conditions (46.2% vs. 7.7% before and after EHF with synbiotics, respectively) (10) which impresses to be in line with our report where only 6.9% of the patients suffered from gastrointestinal infections up to one year of life. Taking these results into account, the low use of healthcare resources observed in our study could also be due to an improvement in the species balance of the intestinal microbiota of patients who received synbiotics which has been previously described (10). It is worth noting that this is only a hypothesis as our study, being a RWE study and not a randomized controlled trial, lacks a control group.

Despite this important limitation, the data obtained in our study together with contributions such as those of Hubbard et al. (10) can help to understand the role of synbiotics in the management of CMPA through changes in the composition of their intestinal microbiota that bring it closer to that of a healthy child.

### 4.5. Preparation and acceptance of an EHF with synbiotics

Although the study had some palatability-related dropouts, a high percentage of infants entering the study protocol appeared to enjoy consuming the formula, as reported by their caregivers. This better palatability of the EHF used in this study may be since it contains whey proteins and lactose, which would result in better palatability than formulas with casein and without lactose (34).

Both an easy preparation and acceptance of an EHF by infants have been reported in the present study and are essential to achieve a good adherence to treatment (35).

### 4.6. Strengths and weaknesses of the present study

The main strength of the study was to be one of the first reports to evaluate the progression of CMPA symptoms treated with an EHF with synbiotics, in a real world setting with a significant sample.

One of the limitations of this study was the relatively high dropout rate, mainly for three reasons: change of treating physician, palatability, or AAF requirement. In relation to palatability, it is important to consider that, since one of the exclusion criteria for the study was having previously consumed hypoallergenic formulas, all the children who entered the protocol had been exclusively breastfed or consuming infant formulas, both more palatable options than an EHF. On the other hand, since those patients who required the consumption of AAF were excluded from the analysis (selective dropout), the results of the study may be slightly affected by attrition bias. Another limitation of the study is that the initial diagnosis of CMPA was based on the clinical judgment of the treating physician, without necessarily being confirmed by a diagnostic oral food challenge. Finally, other weaknesses of the study are that, as being a RWE study, lacks a control group and that its results cannot be extrapolated to other types of synbiotic combinations, as the results are specific for the GOS/FOS and Bifidobacterium breve M-16V combination.

## 5. Conclusions

In this study, the use of an EHF with synbiotics for the management of infants diagnosed with or suspected to have CMPA suggested a good safety profile, an adequate infant growth, and improvement of overall, gastrointestinal, and dermatological symptoms. It also suggested a lower daily frequency of regurgitations and stools, and an improvement in stool consistency, sleeping pattern, and the infant's and the family's quality of life.

## Data Availability

The raw data supporting the conclusions of this article will be made available by the authors, without undue reservation.
