# Supplementary material for: Efficacy of an extensively hydrolyzed formula with the addition of synbiotics in infants with cow's milk protein allergy: a real-world evidence study
Source: Front Allergy. 2023 Oct 9;4:1265083. doi: 10.3389/falgy.2023.1265083 (PMC10591099; doi:10.3389/falgy.2023.1265083)
Supplement: Supplementary file 1 [file Table1.pdf]

Supplementary Material

Efficacy of an Extensively Hydrolyzed Formula with the Addition of Synbiotics in Infants with Cow's Milk Protein Allergy: A Real-world Evidence Study

Soria Ramiro , Del Compare Mónica, Sallaberry Marisa , Martin Graciela , Aprigliano Gustavo, Hermida Verónica, Carosella Mabel, Gruenberg Martín, Monsell Silvana, Micone Paula, Maciero Eugenia, Giglio Norberto*

*** Correspondence:** Corresponding Author: ngiglio@buenosaires.gob.ar

Weight progression (g) in female infants during treatment according to age at admission. Data collected by physicians.

		Day 1	Day 28	1st year
≤3 months	n	22	22	16
	Mean	4544	5467	9201
	95% CI	4122-4965	5008-5926	7942-10460
≥3 months	n	7	7	7
	Mean	7288	7737	9612
	95% CI	6873-7703	7100-8300	8614-10611

Height progression (cm) in female infants during treatment according to age at admission. Data collected by physicians.

		Day 1	Day 28	1st year
≤3 months	n	22	22	16
	Mean	54.4	57.7	73.0
	95% CI	52.8-55.9	56.3-59.1	71.1-74.9
≥3 months	n	7	7	7
	Mean	65.7	66.9	74.7
	95% CI	62.2-69.2	63.5-70.3	72.1-77.4

Head circumference progression (cm) in female infants during treatment according to age at admission. Data collected by physicians.

		Day 1	Day 28	1st year
≤3 months	n	22	22	16
	Mean	37.3	38.7	45.9
	95% CI	36.5-38.2	37.0-40.5	43.8-47.9
≥3 months	n	7	7	7
	Mean	43.1	43.3	46.0
	95% CI	41.5-44.7	42.0-44.3	44.4-47.5

Weight progression (g) in male infants during treatment according to age at admission. Data collected by physicians.

		Day 1	Day 28	1st year
≤3 months	n	18	18	16
	Mean	5170	6211	9851
	95% CI	4784-5556	5773-6648	9133-10570
≥3 months	n	14	14	13
	Mean	7400	8057	9956
	95% CI	6929-7872	7618-8495	9451-10460

Height progression (cm) in male infants during treatment according to age at admission. Data collected by physicians.

		Day 1	Day 28	1st year
≤3 months	n	18	18	16
	Mean	56.0	60.0	74.2
	95% CI	54.6-57.4	58.6-61.3	72.5-75.8
≥3 months	n	14	14	13
	Mean	65.3	67.0	75.5
	95% CI	64.0-66.5	65.7-68.3	73.6-77.3

Head circumference progression (cm) in male infants during treatment according to age at admission. Data collected by physicians.

		Day 1	Day 28	1st year
≤3 months	n	18	18	16
	Mean	38.6	40.4	46.6
	95% CI	37.6-39.5	39.4-41.3	45.6-47.6
≥3 months	n	14	14	13
	Mean	42.5	45.4	45.8
	95% CI	41.9-43.1	42.3-48.5	44.6-47.0
